# 2-(4,5,6,7,8,9-Hexahydro-6a-aza­phenyl­en-2-ylmethyl­ene)indan-1,3-dione

**DOI:** 10.1107/S1600536808016346

**Published:** 2008-06-07

**Authors:** Sergey Belyakov, Valdis Kampars, Pauls J. Pastors, Andrey Tokmakov

**Affiliations:** a Latvian Institute of Organic Synthesis, Riga LV 1006, Latvia; b Department of Materials Science and Applied Chemistry, Riga Technical University, LV 1046, Latvia

## Abstract

The title compound, C_22_H_19_NO_2_, has potential for use as a new nonlinear optical material. Mol­ecules are almost planar. One C atom of the heterocyclic ring system is disordered over two positions; the site occupancy factors are 0.6 and 0.4.

## Related literature

For related literature, see: Honda *et al.* (1996 ▶); Allen (2002 ▶).
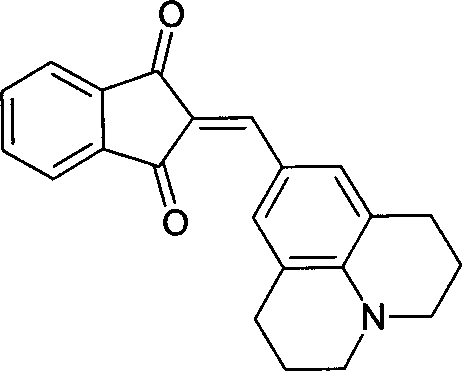


## Experimental

### 

#### Crystal data

C_22_H_19_NO_2_
*M* *_r_* = 329.38
Monoclinic, 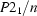

*a* = 8.5125 (2) Å
*b* = 19.2973 (5) Å
*c* = 10.4969 (3) Å
β = 109.5301 (10)°
*V* = 1625.10 (7) Å^3^
*Z* = 4
Mo *K*α radiation
μ = 0.09 mm^−1^
*T* = 293 (2) K
0.26 × 0.19 × 0.04 mm

#### Data collection

Nonius KappaCCD diffractometer
Absorption correction: none
6190 measured reflections
3685 independent reflections
2852 reflections with *I* > 2σ(*I*)
*R*_int_ = 0.021

#### Refinement

*R*[*F* ^2^ > 2σ(*F* ^2^)] = 0.055
*wR*(*F* ^2^) = 0.155
*S* = 1.01
3685 reflections
255 parameters
H-atom parameters constrained
Δρ_max_ = 0.27 e Å^−3^
Δρ_min_ = −0.39 e Å^−3^

### 

Data collection: *KappaCCD Server Software* (Nonius, 1999 ▶); cell refinement: *KappaCCD Server Software*; data reduction: *DENZO* and *SCALEPACK* (Otwinowski & Minor, 1997 ▶); program(s) used to solve structure: *maXus* (Mackay *et al.*, 1999 ▶) and *SIR92* (Altomare *et al.*, 1994 ▶); program(s) used to refine structure: *maXus* and *SHELXL97* (Sheldrick, 2008 ▶); molecular graphics: *ORTEP-3* (Farrugia, 1997 ▶); software used to prepare material for publication: *SHELXL97*.

## Supplementary Material

Crystal structure: contains datablocks global, I. DOI: 10.1107/S1600536808016346/rk2090sup1.cif

Structure factors: contains datablocks I. DOI: 10.1107/S1600536808016346/rk2090Isup2.hkl

Additional supplementary materials:  crystallographic information; 3D view; checkCIF report

## Figures and Tables

**Fig. 1.**
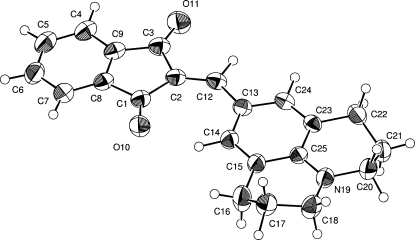
The molecular structure of (I) with the atom numbering scheme. The displacement ellipsoids are showed at 50% probability level. H atoms are represented by spheres of arbitrary radii. Only major fragnent are presented for clarity.

**Fig. 2.**
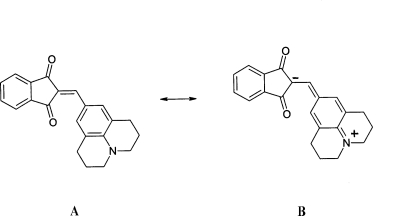
Two mesomeric forms for molecular structure of I.

**Fig. 3.**
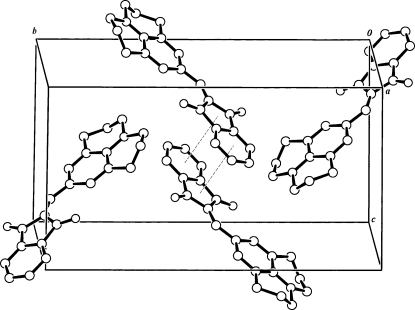
Perspective view of the molecular packing for I, showing the stacking interactions betwee indandione systems.

## supplementary crystallographic information

### Comment

The molecular structure of the title compound, C_22_H_19_NO_2_, (I), with atomic numbering scheme and thermal ellipsoids is presented in Fig. 1. The indandione fragment geometry is usual. The aromatic C14-C15 and C23-C24 bonds are shorter than other aromatic bonds in yulolidine system, indicating the quinoid character. Thus, presenting schematically the structure of I as two mesomeric forms (A or B) one can infer that the specific weight of the ionic form of B is increased (see Fig. 2). Therefore, the deep coloration occurs for the crystals I. A search of the Cambridge Structural Database (CSD, Version 5.29, November 2007; Allen, 2002) indicates that there are only 26 entries containing yulolidine fragments. For the title compound there is the disorder of crystal structure analogously to the crystal structure of "Coumarin 106" (Honda *et al.*,  1996). In the yulolidine system the C17 atom is disordered and the site occupancies were initially refined then fixed at 0.6 and 0.4 for C17 and C17', respectively, in the final refinement. Atoms C17 and C17' are located on the opposite sides of the least-squares plane of the molecule. The atoms C17, C17' and C21 deviate from the molecule plane on 0.597 (4), -0.288 (6) and -0.527 (2)Å, respectively.

The packing diagram of the molecules is given in Fig. 3. The moderate π-π-stacking interaction in the crystal structure of (I) is between paris of inversion-related indandione systems. The five-membered cycle overlaps with the benzene ring of indandione; the centroids of these rings are separated by 3.509 (3)Å, but the distance between planes of these indandione systems is 3.435 (3)Å.

### Experimental

A mixture of indan-1,3-dione, (0.44 g, 3.0 mmole), yulolidine-9-carbaldehyde (0.62 g, 3.1 mmole) of and 30 ml of absolute ethanol was boiled for 15 minutes, cooled to room temperature and filtered. Deep red crystals of I with metallic sheen were obtained after recrystallyzation from ethanol. *M.p.* is 504 K (decomp.); Yield 83%. Analysis calculated for C_22_H_19_NO_2_: C 80.22, H 5.81, N 4.25%; found: C 80.07, H 5.43, N 4.30%.

### Refinement

The H atoms were place in geometrically idealized positions, with C–H distances of 0.93Å for aromatic H atoms and 0.96Å for other H-atoms. All H atoms were refined riding on their attached C atoms, with *U*_iso_ values equal to 1.2 times the *U*_eq_ values of the parent atoms.

### Crystal data

**Table:**

C_22_H_19_NO_2_	*F*_000_ = 696
*M**_r_* = 329.38	*D*_x_ = 1.346 Mg m^−^^3^
Monoclinic, *P*2_1_/*n*	Mo *K*α radiation λ = 0.71073 Å
Hall symbol: -P 2yn	Cell parameters from 6190 reflections
*a* = 8.5125 (2) Å	θ = 2.1–27.5º
*b* = 19.2973 (5) Å	µ = 0.09 mm^−^^1^
*c* = 10.4969 (3) Å	*T* = 293 (2) K
β = 109.5301 (10)º	Plate, red
*V* = 1625.10 (7) Å^3^	0.26 × 0.19 × 0.04 mm
*Z* = 4	

### Data collection

**Table:**

Nonius KappaCCD diffractometer	2852 reflections with *I* > 2σ(*I*)
Radiation source: fine-focus sealed tube	*R*_int_ = 0.021
Monochromator: graphite	θ_max_ = 27.5º
*T* = 293(2) K	θ_min_ = 2.1º
φ and ω scans	*h* = −11→11
Absorption correction: none	*k* = −24→23
6190 measured reflections	*l* = −13→13
3685 independent reflections	

### Refinement

**Table:**

Refinement on *F*^2^	Secondary atom site location: Difmap
Least-squares matrix: full	Hydrogen site location: Geom
*R*[*F*^2^ > 2σ(*F*^2^)] = 0.055	H-atom parameters constrained
*wR*(*F*^2^) = 0.155	Calculated *w* = 1/[σ^2^(*F*_o_^2^) + (0.0792*P*)^2^ + 0.4361*P*] where *P* = (*F*_o_^2^ + 2*F*_c_^2^)/3 ?
*S* = 1.01	(Δ/σ)_max_ = 0.008
3685 reflections	Δρ_max_ = 0.27 e Å^−^^3^
255 parameters	Δρ_min_ = −0.39 e Å^−^^3^
Primary atom site location: Direct	Extinction correction: none

### Special details

**Table:**

Geometry. All s.u.'s (except the s.u. in the dihedral angle between two l.s. planes) are estimated using the full covariance matrix. The cell s.u.'s are taken into account individually in the estimation of s.u.'s in distances, angles and torsion angles; correlations between s.u.'s in cell parameters are only used when they are defined by crystal symmetry. An approximate (isotropic) treatment of cell s.u.'s is used for estimating s.u.'s involving l.s. planes.
Refinement. Refinement of *F*^2^ against ALL reflections. The weighted *R*-factor *wR* and goodness of fit *S* are based on *F*^2^, conventional *R*-factors *R* are based on *F*, with *F* set to zero for negative *F*^2^. The threshold expression of *F*^2^ > σ(*F*^2^) is used only for calculating *R*-factors(gt) *etc*. and is not relevant to the choice of reflections for refinement. *R*-factors based on *F*^2^ are statistically about twice as large as those based on *F*, and *R*-factors based on ALL data will beeven larger.

### Fractional atomic coordinates and isotropic or equivalent isotropic displacement parameters (Å^2^)

**Table:**

	*x*	*y*	*z*	*U*_iso_*/*U*_eq_	Occ. (<1)
C1	1.0787 (2)	0.02825 (8)	0.26816 (16)	0.0432 (4)	
C2	0.9234 (2)	−0.00221 (8)	0.27606 (15)	0.0423 (4)	
C3	0.8928 (2)	−0.06707 (8)	0.19540 (17)	0.0475 (4)	
C4	1.0528 (2)	−0.12254 (9)	0.04817 (18)	0.0539 (4)	
H4	0.9807	−0.1600	0.0197	0.065*	
C5	1.1874 (2)	−0.11410 (10)	0.00365 (19)	0.0579 (5)	
H5	1.2054	−0.1463	−0.0560	0.069*	
C6	1.2957 (2)	−0.05867 (10)	0.04616 (19)	0.0585 (5)	
H6	1.3854	−0.0542	0.0149	0.070*	
C7	1.2720 (2)	−0.00962 (9)	0.13495 (18)	0.0526 (4)	
H7	1.3447	0.0276	0.1640	0.063*	
C8	1.1372 (2)	−0.01783 (8)	0.17868 (15)	0.0432 (4)	
C9	1.0285 (2)	−0.07358 (8)	0.13643 (16)	0.0447 (4)	
O10	1.15210 (15)	0.08098 (6)	0.32016 (13)	0.0576 (4)	
O11	0.77589 (18)	−0.10689 (7)	0.17663 (15)	0.0686 (4)	
C12	0.8117 (2)	0.01796 (8)	0.33618 (15)	0.0439 (4)	
H12	0.7261	−0.0140	0.3238	0.053*	
C13	0.79509 (19)	0.07662 (8)	0.41372 (15)	0.0411 (4)	
C14	0.9044 (2)	0.13387 (8)	0.44714 (17)	0.0462 (4)	
H14	0.9949	0.1349	0.4163	0.055*	
C15	0.8815 (2)	0.18804 (8)	0.52349 (18)	0.0471 (4)	
C16	0.9979 (3)	0.24908 (11)	0.5566 (3)	0.0806 (7)	
H16A	1.1051	0.2352	0.5539	0.097*	0.60
H16B	0.9532	0.2850	0.4912	0.097*	0.60
H16C	1.1060	0.2347	0.6147	0.097*	0.40
H16D	1.0066	0.2676	0.4744	0.097*	0.40
C17	1.0199 (4)	0.27643 (19)	0.6845 (4)	0.0554 (7)	0.60
H17A	1.0823	0.2443	0.7524	0.067*	0.60
H17B	1.0798	0.3194	0.6949	0.067*	0.60
C17'	0.9495 (7)	0.3072 (3)	0.6257 (6)	0.0557 (11)	0.40
H17C	1.0477	0.3309	0.6812	0.067*	0.40
H17D	0.8808	0.3388	0.5597	0.067*	0.40
C18	0.8574 (2)	0.29182 (10)	0.7109 (2)	0.0615 (5)	
H18A	0.8169	0.3357	0.6700	0.074*	0.60
H18B	0.8839	0.2952	0.8071	0.074*	0.60
H18C	0.8064	0.3340	0.7258	0.074*	0.40
H18D	0.9360	0.2761	0.7949	0.074*	0.40
N19	0.72946 (17)	0.23902 (7)	0.65742 (14)	0.0466 (3)	
C20	0.5980 (2)	0.23753 (10)	0.71759 (18)	0.0531 (4)	
H20A	0.5683	0.2842	0.7317	0.064*	
H20B	0.6392	0.2152	0.8042	0.064*	
C21	0.4455 (2)	0.19991 (10)	0.6302 (2)	0.0557 (5)	
H21A	0.3947	0.2258	0.5486	0.067*	
H21B	0.3663	0.1964	0.6771	0.067*	
C22	0.4894 (2)	0.12830 (9)	0.59526 (18)	0.0505 (4)	
H22A	0.5184	0.0996	0.6744	0.061*	
H22B	0.3941	0.1081	0.5283	0.061*	
C23	0.63360 (19)	0.13043 (8)	0.54173 (15)	0.0405 (3)	
C24	0.65899 (19)	0.07823 (8)	0.46198 (16)	0.0429 (4)	
H24	0.5825	0.0420	0.4385	0.051*	
C25	0.74777 (18)	0.18666 (8)	0.57636 (15)	0.0386 (3)	

### Atomic displacement parameters (Å^2^)

**Table:**

	*U*^11^	*U*^22^	*U*^33^	*U*^12^	*U*^13^	*U*^23^
C1	0.0486 (8)	0.0375 (8)	0.0416 (8)	0.0002 (6)	0.0127 (6)	−0.0002 (6)
C2	0.0505 (9)	0.0344 (7)	0.0408 (7)	−0.0031 (6)	0.0134 (7)	−0.0001 (6)
C3	0.0559 (9)	0.0382 (8)	0.0465 (9)	−0.0039 (7)	0.0148 (7)	−0.0019 (7)
C4	0.0628 (11)	0.0430 (9)	0.0532 (10)	0.0064 (8)	0.0158 (8)	−0.0053 (7)
C5	0.0709 (12)	0.0508 (10)	0.0529 (10)	0.0159 (9)	0.0218 (9)	−0.0032 (8)
C6	0.0605 (11)	0.0585 (11)	0.0620 (11)	0.0167 (9)	0.0278 (9)	0.0065 (9)
C7	0.0525 (10)	0.0480 (9)	0.0580 (10)	0.0035 (7)	0.0194 (8)	0.0029 (8)
C8	0.0482 (8)	0.0375 (8)	0.0405 (8)	0.0063 (6)	0.0104 (7)	0.0032 (6)
C9	0.0530 (9)	0.0365 (8)	0.0400 (8)	0.0052 (7)	0.0093 (7)	0.0008 (6)
O10	0.0591 (7)	0.0499 (7)	0.0697 (8)	−0.0157 (6)	0.0291 (6)	−0.0182 (6)
O11	0.0757 (9)	0.0525 (8)	0.0845 (10)	−0.0240 (7)	0.0358 (7)	−0.0233 (7)
C12	0.0513 (9)	0.0370 (8)	0.0427 (8)	−0.0083 (6)	0.0146 (7)	−0.0002 (6)
C13	0.0462 (8)	0.0368 (7)	0.0406 (8)	−0.0024 (6)	0.0148 (6)	0.0013 (6)
C14	0.0438 (8)	0.0418 (8)	0.0577 (10)	−0.0031 (7)	0.0235 (7)	−0.0059 (7)
C15	0.0416 (8)	0.0404 (8)	0.0630 (10)	−0.0048 (6)	0.0225 (7)	−0.0073 (7)
C16	0.0712 (13)	0.0601 (12)	0.135 (2)	−0.0293 (10)	0.0664 (14)	−0.0441 (13)
C17	0.0490 (16)	0.0517 (18)	0.0645 (19)	−0.0096 (14)	0.0176 (15)	−0.0153 (16)
C17'	0.058 (3)	0.044 (3)	0.068 (3)	−0.010 (2)	0.025 (2)	−0.007 (2)
C18	0.0574 (10)	0.0550 (11)	0.0747 (12)	−0.0070 (8)	0.0258 (9)	−0.0237 (9)
N19	0.0465 (7)	0.0460 (8)	0.0511 (8)	−0.0001 (6)	0.0211 (6)	−0.0054 (6)
C20	0.0578 (10)	0.0537 (10)	0.0554 (10)	0.0087 (8)	0.0291 (8)	0.0006 (8)
C21	0.0482 (9)	0.0641 (11)	0.0634 (11)	0.0081 (8)	0.0299 (8)	0.0103 (9)
C22	0.0479 (9)	0.0550 (10)	0.0527 (9)	−0.0050 (7)	0.0223 (7)	0.0063 (8)
C23	0.0393 (7)	0.0432 (8)	0.0384 (7)	−0.0009 (6)	0.0122 (6)	0.0073 (6)
C24	0.0455 (8)	0.0393 (8)	0.0433 (8)	−0.0078 (6)	0.0140 (7)	0.0023 (6)
C25	0.0380 (7)	0.0376 (7)	0.0390 (7)	0.0029 (6)	0.0113 (6)	0.0033 (6)

### Geometric parameters (Å, °)

**Table:**

C1—O10	1.2231 (19)	C17—C18	1.528 (4)
C1—C2	1.474 (2)	C17—H16C	1.4431
C1—C8	1.494 (2)	C17—H17A	0.9600
C2—C12	1.362 (2)	C17—H17B	0.9600
C2—C3	1.485 (2)	C17—H17C	1.0804
C3—O11	1.220 (2)	C17—H18D	1.5509
C3—C9	1.487 (2)	C17'—C18	1.405 (5)
C4—C5	1.384 (3)	C17'—H16B	1.4854
C4—C9	1.386 (2)	C17'—H17B	1.1288
C4—H4	0.9300	C17'—H17C	0.9599
C5—C6	1.385 (3)	C17'—H17D	0.9600
C5—H5	0.9300	C17'—H18A	1.4642
C6—C7	1.390 (3)	C18—N19	1.460 (2)
C6—H6	0.9300	C18—H18A	0.9600
C7—C8	1.380 (2)	C18—H18B	0.9600
C7—H7	0.9300	C18—H18C	0.9600
C8—C9	1.391 (2)	C18—H18D	0.9600
C12—C13	1.429 (2)	N19—C25	1.363 (2)
C12—H12	0.9300	N19—C20	1.458 (2)
C13—C14	1.411 (2)	C20—C21	1.503 (3)
C13—C24	1.412 (2)	C20—H20A	0.9600
C14—C15	1.370 (2)	C20—H20B	0.9600
C14—H14	0.9300	C21—C22	1.509 (3)
C15—C25	1.423 (2)	C21—H21A	0.9600
C15—C16	1.503 (2)	C21—H21B	0.9600
C16—C17	1.396 (4)	C22—C23	1.512 (2)
C16—C17'	1.468 (5)	C22—H22A	0.9600
C16—H16A	0.9600	C22—H22B	0.9600
C16—H16B	0.9601	C23—C24	1.372 (2)
C16—H16C	0.9600	C23—C25	1.420 (2)
C16—H16D	0.9600	C24—H24	0.9300
C17—C17'	0.920 (6)		
			
O10—C1—C2	129.84 (15)	H16C—C17—H18D	144.7
O10—C1—C8	123.21 (15)	H17A—C17—H18D	73.8
C2—C1—C8	106.95 (13)	H17B—C17—H18D	106.2
C12—C2—C1	133.65 (15)	H17C—C17—H18D	100.9
C12—C2—C3	119.24 (14)	C17—C17'—C18	79.2 (4)
C1—C2—C3	107.09 (14)	C17—C17'—C16	67.1 (4)
O11—C3—C9	125.77 (15)	C18—C17'—C16	117.5 (4)
O11—C3—C2	126.97 (17)	C17—C17'—H16B	103.6
C9—C3—C2	107.21 (13)	C18—C17'—H16B	137.5
C5—C4—C9	118.09 (17)	C16—C17'—H16B	37.9
C5—C4—H4	121.0	C17—C17'—H17B	54.7
C9—C4—H4	121.0	C18—C17'—H17B	105.8
C6—C5—C4	121.28 (17)	C16—C17'—H17B	95.7
C6—C5—H5	119.4	H16B—C17'—H17B	110.1
C4—C5—H5	119.4	C17—C17'—H17C	70.1
C5—C6—C7	120.78 (18)	C18—C17'—H17C	105.8
C5—C6—H6	119.6	C16—C17'—H17C	109.4
C7—C6—H6	119.6	H16B—C17'—H17C	115.0
C8—C7—C6	117.91 (17)	H17B—C17'—H17C	16.5
C8—C7—H7	121.0	C17—C17'—H17D	175.6
C6—C7—H7	121.0	C18—C17'—H17D	105.0
C7—C8—C9	121.41 (15)	C16—C17'—H17D	109.4
C7—C8—C1	129.00 (15)	H16B—C17'—H17D	72.4
C9—C8—C1	109.55 (14)	H17B—C17'—H17D	124.3
C8—C9—C4	120.53 (17)	H17C—C17'—H17D	109.5
C8—C9—C3	109.16 (14)	C17—C17'—H18A	115.3
C4—C9—C3	130.25 (16)	C18—C17'—H18A	39.0
C2—C12—C13	135.06 (15)	C16—C17'—H18A	144.8
C2—C12—H12	112.5	H16B—C17'—H18A	132.4
C13—C12—H12	112.5	H17B—C17'—H18A	114.4
C14—C13—C24	116.46 (14)	H17C—C17'—H18A	103.7
C14—C13—C12	125.41 (14)	H17D—C17'—H18A	69.1
C24—C13—C12	118.14 (14)	C17'—C18—N19	114.0 (2)
C15—C14—C13	122.13 (15)	C17'—C18—C17	36.3 (2)
C15—C14—H14	118.9	N19—C18—C17	113.50 (17)
C13—C14—H14	118.9	C17'—C18—H18A	73.8
C14—C15—C25	120.17 (14)	N19—C18—H18A	109.5
C14—C15—C16	121.40 (16)	C17—C18—H18A	107.6
C25—C15—C16	118.42 (15)	C17'—C18—H18B	132.1
C17—C16—C17'	37.4 (2)	N19—C18—H18B	109.5
C17—C16—C15	112.6 (2)	C17—C18—H18B	107.2
C17'—C16—C15	116.2 (2)	H18A—C18—H18B	109.5
C17—C16—H16A	108.1	C17'—C18—H18C	107.7
C17'—C16—H16A	130.8	N19—C18—H18C	109.5
C15—C16—H16A	109.6	C17—C18—H18C	132.9
C17—C16—H16B	107.9	H18A—C18—H18C	37.8
C17'—C16—H16B	72.0	H18B—C18—H18C	74.4
C15—C16—H16B	109.1	C17'—C18—H18D	106.6
H16A—C16—H16B	109.5	N19—C18—H18D	109.5
C17—C16—H16C	72.9	C17—C18—H18D	73.1
C17'—C16—H16C	106.0	H18A—C18—H18D	136.5
C15—C16—H16C	109.8	H18B—C18—H18D	37.8
H16A—C16—H16C	38.7	H18C—C18—H18D	109.5
H16B—C16—H16C	136.9	C25—N19—C20	121.36 (14)
C17—C16—H16D	134.2	C25—N19—C18	122.09 (14)
C17'—C16—H16D	106.1	C20—N19—C18	115.42 (14)
C15—C16—H16D	109.2	N19—C20—C21	112.15 (14)
H16A—C16—H16D	73.6	N19—C20—H20A	109.2
H16B—C16—H16D	38.7	C21—C20—H20A	109.2
H16C—C16—H16D	109.5	N19—C20—H20B	109.2
C17'—C17—C16	75.6 (4)	C21—C20—H20B	109.2
C17'—C17—C18	64.5 (4)	H20A—C20—H20B	107.9
C16—C17—C18	114.2 (2)	C20—C21—C22	110.93 (14)
C17'—C17—H16C	110.3	C20—C21—H21A	109.5
C16—C17—H16C	39.5	C22—C21—H21A	109.5
C18—C17—H16C	147.7	C20—C21—H21B	109.5
C17'—C17—H17A	172.0	C22—C21—H21B	109.5
C16—C17—H17A	109.5	H21A—C21—H21B	108.0
C18—C17—H17A	107.5	C23—C22—C21	111.32 (14)
H16C—C17—H17A	76.6	C23—C22—H22A	109.4
C17'—C17—H17B	73.7	C21—C22—H22A	109.4
C16—C17—H17B	109.3	C23—C22—H22B	109.4
C18—C17—H17B	106.9	C21—C22—H22B	109.4
H16C—C17—H17B	101.5	H22A—C22—H22B	108.0
H17A—C17—H17B	109.5	C24—C23—C25	118.88 (14)
C17'—C17—H17C	56.7	C24—C23—C22	121.31 (14)
C16—C17—H17C	107.3	C25—C23—C22	119.79 (14)
C18—C17—H17C	92.2	C23—C24—C13	123.41 (14)
H16C—C17—H17C	111.9	C23—C24—H24	118.3
H17A—C17—H17C	125.4	C13—C24—H24	118.3
H17B—C17—H17C	18.2	N19—C25—C15	120.18 (14)
C17'—C17—H18D	98.3	N19—C25—C23	120.98 (14)
C16—C17—H18D	140.3	C15—C25—C23	118.83 (14)
C18—C17—H18D	36.3		
